# Depressive delusions^1^

**DOI:** 10.4013/fsu.2016.172.14

**Published:** 2016-10-04

**Authors:** Magdalena Antrobus, Lisa Bortolotti

**Affiliations:** 2 Philosophy Department, University of Birmingham. Edgbaston B15 2TT, Birmingham, United Kingdom

**Keywords:** depression, delusion, schema, learning, anxiety, cognitive dissonance, epistemic innocence

## Abstract

In this paper we have two main aims. First, we present an account of mood-congruent delusions in depression (hereafter, *depressive delusions*). We propose that depressive delusions constitute acknowledgements of self-related beliefs acquired as a result of a negatively biased learning process. Second, we argue that depressive delusions have the potential for psychological and epistemic benefits despite their obvious epistemic and psychological costs. We suggest that depressive delusions play an important role in preserving a person’s overall coherence and narrative identity at a critical time, and thus can be regarded as *epistemically innocent*.

**Resumo:**

Nesse artigo temos dois objetivos principais: primeiro, sugerimos um modo de compreender delírios congruentes com o humor na depressão (doravante, delírios depressivos) e como estes se distinguem de delírios na esquizofrenia; segundo, investigamos se delírios depressivos podem trazer benefícios epistêmicos e psicológicos. Propomos que delírios depressivos emergem como resultado de um processo de aprendizado negativamente enviesado e constituem um reconhecimento de informação sobre o sujeito que é adquirido como resultado desse processo de aprendizado. Argumentamos que delírios depressivos desempenham um importante papel na preservação da coerência e da identidade narrativa de agentes em momentos de crise.

**Palavras-chave**

depressão, delírio, esquema, aprendizagem, ansiedade, dissonância cognitiva, inocência epistêmica.

## Introduction

What are delusions? It is common to define delusions as implausible beliefs that are held with conviction but for which there is little empirical support.

Delusion. A false belief based on incorrect inference about external reality that is firmly sustained despite what almost everyone else believes and despite what constitutes incontrovertible and obvious proof or evidence to the contrary. The belief is not one ordinarily accepted by other members of the person’s culture or subculture (e.g., it is not an article of religious faith). When a false belief involves a value judgment, it is regarded as a delusion only when the judgment is so extreme as to defy credibility (APA, 2013, p. 819).

For instance, Leslie who has delusions of persecution may come to believe that other people intend to cause her harm. Such a delusion has epistemic and psychological costs for Leslie. By interpreting many of her experiences in the light of her delusions, Leslie misrepresents the intentions of her family members and colleagues, and comes to regard their behaviour towards her as more hostile than it really is. Persecutory delusions also have a negative effect on Leslie’s well-being by contributing to her increasing distress and anxiety. Apart from persecution, common delusional content found in depression involves guilt, illness, and loss of one’s financial integrity.

Delusions of guilt articulate the concern to have lost one’s moral integrity, that is, one’s commitment to be there for the others. Delusions of illness express the concern to have lost one’s physical integrity, the worry not to be autonomous and to be a burden for the others. Finally, delusions of ruin are about the concern to have lost one’s financial integrity, thus, the worry to get the others involved in one’s bankruptcy (Stanghellini and Raballo, 2015, p. 174).

Delusions in general, and delusions of persecution in particular, have been the object of extensive psychological and phenomenological research (Freeman *et al*., 2001; Boyd and Gumley, 2007; Campbell and Morrison, 2007; Freeman, 2007; Freeman *et al*., 2008), and they have recently become a subject of philosophical investigation as well (Bortolotti, 2009; Radden, 2010; Gerrans, 2014). Most of the literature concentrates on delusional experience as part of schizophrenia or of the wider category of psychosis. But some of the recent research on depression (e.g., by Stanghellini and Raballo, 2015) suggests that delusions also emerge in the context of major depressive disorders and present different characteristics from those found in psychosis.

In this paper we are interested in the nature of depressive delusions and ask whether they have some benefits despite their obvious epistemic and psychological costs. Here is our plan. In the section “Schizophrenic delusions versus depressive delusions”, we describe what the existing literature identifies as different features in schizophrenic and depressive delusions. In the section “Learning about the self in depression”, we consider cognitive distortions in the learning processes that may contribute to severe depression. In “A hypothesis about the development of depressive delusions”, we argue that the content of depressive delusions is related to the content of those beliefs that people acquire about themselves due to negatively biased learning. In the fifth section, “Psychological and epistemic benefits of depressive delusions”, we suggest that depressive delusions are acknowledgments of previously acquired self-related beliefs and we maintain that, despite its costs, the adoption of depressive delusions also has some psychological and epistemic benefits. Depressive delusions can reduce anxiety caused by inconsistent cognitions, and can also be viewed as *epistemically innocent*, where epistemic innocence is a property of epistemically costly cognitions that have significant epistemic benefits and whose benefits cannot be easily attained via less costly cognitions.

## Schizophrenic delusions versus depressive delusions

A severe form of depression, sometimes called *psychotic depression* or *depressive psychosis*, is a major depressive episode that is accompanied by psychotic symptoms such as delusions and hallucinations (Hales and Yudofsky, 2003; Gerretsen *et al*., 2015) as well as by intense depressive symptoms, including very low mood, lack of interest in everyday matters, problems with sleeping, feelings of guilt, and obsessive self-accusations.

The vast majority of delusions appearing in severe depression are *mood-congruent*, which means that their content matches the mood experienced by the person (Hales and Yudofsky, 2003). According to the Diagnostic and Statistical Manual of Mental Disorders (APA, 2013), common themes of depressive delusions are persecution, guilt, punishment, personal inadequacy, or disease, with half of the affected people experiencing delusions with more than one theme. It has been estimated that depression involving delusions occurs in 16% to 54% of people suffering from depression (Rothschild *et al*., 1999).

It is a matter of dispute whether depression involving delusions is a severe form of unipolar depression (sometimes also referred to as *melancholia*) or a condition that falls into the broader category of psychosis (see for instance: Frances *et al*., 1981; Parker *et al*., 1992). One argument for the latter hypothesis comes from empirical research showing that people suffering from these severe forms of depression do not get better with antidepressant medication alone (Brown *et al*., 1982). Other researchers (e.g., Nelson and Bowers, 1978), however, argue on the basis of clinical studies that depression involving delusions should be understood as a sub-type of major depressive disorder and that it is continuous with, and not detached from, other forms of depressive illness. Stanghellini and Raballo (2015) provide further support for this claim and point to several differences between *schizophrenic* and *depressive* delusions. According to them, delusions that emerge in schizophrenia take the form of a revelation by uncovering some *new* content, *unfamiliar* to the person, whereas delusions that emerge in depression confirm self-related information that is *already known* and *familiar*.

This seems to be an important point. According to Stanghellini and Raballo (2015), the experience of schizophrenic delusions amounts to being struck with a ‘something’ that opens up a new meaning, a new identity, or a new understanding of the world that is deeper and more personal. This new awareness manifests as a sudden disclosure (Stanghellini and Ballerini, 1992), or as a kind of aesthetic experience (Parnas, 2013). The revelation follows a phase of uncertainty and tension and signals a radical change. The revelation is the ‘dawn of a new reality’ (Stanghellini and Rosfort, 2013).

This feature of schizophrenic delusions has been captured by several other authors in the classic literature on schizophrenia. Karl Jaspers (1963) spoke of how endorsing the delusional content puts an end to a long period of uncertainty.

This general delusional atmosphere with all its vagueness of content must be unbearable. Patients obviously suffer terribly under it and to reach some definite idea at last is like being relieved from some enormous burden […] The achievement brings strength and comfort […] No dread is worse than that of danger unknown (Jaspers, 1963, p. 98).

And Klaus Conrad introduced the concept of ‘apophany,’ the manifestation of a meaningfulness that was previously hidden. Here is a case study on which Conrad relied for his analysis of delusions as a revelation:

Due to possible psychosis, a 32-year-old first-class private, Karl B., was brought to Dr Conrad’s hospital. In his interview with Conrad, the patient reports that “everything begins” one morning as his unit breaks to leave camp. When the sergeant asks him for the key to his quarters, it is suddenly clear to him that it is a ploy to “test” him. While departing in the bus, he notices that his comrades are behaving strangely: They know something that he is not supposed to know. One of his comrades asks “conspicuously” if he has any bread. At midday, they arrive in a town to relieve units positioned there. A few in his company are charged with finding quarters for the rest of them. This is only a ruse for the few to receive instructions in how to deal with him while he waits with the others in the motor coach. One after another, groups of men leave the coach only for the others to return. “It is clear that they are all receiving their instructions” about him. The patient is unable to explain how he sees this. He simply “sees it” (Mishara, 2010, p. 9, our emphasis).

In schizophrenic delusions new content is revealed. People affected by schizophrenia often describe the adoption of the delusion as a *discovery*, such as the discovery of the true meaning of life, or of a new purpose for humanity (Stanghellini and Raballo, 2015, p. 173), although delusions can and often do incorporate aspects of the person’s everyday reality and past experience (see for instance Parnas and Sass, 2001; Freeman *et al*., 2008).

Do depressive delusions share the same features as schizophrenic delusions? Not quite. There is wide agreement that depressive delusions *confirm* the person’s previously acquired self-related beliefs and her feelings (see Kraus, 1983), instead of manifesting as a revelation or as the discovery of something new. Delusions of guilt in severe depression may validate a feeling of guilt and confirm the person’s conviction that she has done something wrong. The idea that depressive delusions are preceded by moods and beliefs that are congruent with their content has been put forward in several contexts. For instance, Matthew Ratcliffe (2008), who adopts the Heidegger-inspired term ‘existential feelings’ to describe what the clinical literature refers to as ‘moods,’ maintains that existential feelings provide the basis for further experience and thought, including delusional thoughts. Josef Parnas (2013) suggests that depressive delusions deal with the worldly affairs in which patients are engaged and for which they seek evidence. As depressive delusions have a direct link to previous experience and are about everyday feelings, Parnas calls them ‘empirical,’ to distinguish them from the more ‘metaphysical’ delusions that give an insight into the nature of reality and are typical of schizophrenia.

## Learning about the self in depression

According to Swiss developmental psychologist Jean Piaget (1977), there are different stages of cognitive development explaining how children acquire and process knowledge. Some of Piaget’s findings have been used to explain cognitive abnormalities in mental disorders such as schizophrenia and depression (e.g., Beck *et al*., 1979). Here we are going to refer to one of Piaget’s concepts, the *equilibration of cognitive structures* model (hereafter, *ECS*) in order to shed light on the mechanisms involved in the acquisition of self-related beliefs in depression.

The central concept in the ECS model is that of *a schema*. A schema can be defined as a set of linked mental representations of the world, which people use both to understand new situations and to respond to them. In other words, the schema is a stored representation of a pattern of behaviour. For example, a person might have a schema about buying a meal in a restaurant, which includes looking at a menu, ordering food, eating, and paying the bill. Whenever a person with this schema finds herself in a restaurant, she retrieves the schema from memory, and applies it to her situation. Piaget described how schemata were acquired and modified.

According to the ECS model, when the person’s existing schemata can explain what she experiences, then we have a state of *equilibrium*, or cognitive balance. Two key processes are required for maintaining cognitive balance: *assimilation*, which is using an existing schema to deal with a new object or situation; and *accommodation*, which happens when the existing schema does not correctly apply to a new situation and needs to be modified as a result. A correct development of cognitive structures (also called ‘adaptation’) requires effective implementation of both processes, assimilation and accommodation. If one of the two processes does not perform its function, the other one attempts to compensate to ensure adaptation. The main aim is to avoid conflicting cognitions and maintain coherence. Processes such as assimilation and accommodation serve this purpose.

When equilibrium cannot be achieved, increased levels of stress and anxiety are experienced. Consider the following example:

Raymond, seeing a panther for the first time, says, ‘look, there is a black leopard.’ He has fit the new animal into an existing mental structure because he is already familiar with leopards and just assumes that the panther is a leopard of a different colour. He is using assimilation, making the new information fit into what he already knows. Monique, however, has never visited a zoo and has seen some wild animals only on television or in books. Seeing unfamiliar llamas she considers what these might be. They resemble horses, but Monique immediately dismisses this category because she knows that horses have smooth hair and shorter necks. She also dismisses camels because she knows they have humps on their backs. She decides finally that this must be an animal she does not know and asks the teacher what it is. Monique is using accommodation, creating a new concept into which this new information can be fitted (Essa, 2012, p. 117).

Seeing llamas for the first time and not being able to categorise them causes a state of *cognitive dissonance*. This state resembles Piaget’s lack of equilibrium and is defined as the mental stress or discomfort experienced by an individual who holds two or more contradictory beliefs, ideas, or values at the same time; performs an action that is contradictory to one or more beliefs, ideas, or values; or is confronted by new information that conflicts with existing beliefs, ideas, or values (Festinger, 1957, 1962).

The theory of cognitive dissonance helps us understand the extent to which people strive for coherence. When people experience inconsistent cognitions, they become psychologically uncomfortable and are motivated to reduce the inconsistency—as well as actively avoid situations and information likely to increase it (Festinger, 1957). When people are confronted with information that conflicts with their previous beliefs and cannot change such beliefs, they attempt to restore consistency in other ways, for instance by reinterpreting their experience, rejecting the new information, or seeking additional support for the previous beliefs from other people who share such beliefs (Harmon-Jones, 2002). There is evidence suggesting that people who remain in a state of cognitive dissonance for a prolonged period of time experience increased levels of anxiety and symptoms resembling those of post-traumatic stress disorder (Eysenck, 2013).

The hypothesis we wish to explore and ultimately defend is that people with severe depression acquire increasingly negative beliefs about themselves because the process by which they acquire self-related information is disrupted. The balance between assimilation and accommodation is compromised in that people with depression become increasingly less able to use accommodation as a way of processing new self-related information. Instead, they distort the content of newly acquired information in order to assimilate it successfully and making it consistent with their previous beliefs.

As we saw, people use schemata to understand the world, including themselves, and to learn from their experiences. Schemata have been shaped by past experiences, memories, social interactions, and conscious reflection, as well as by a number of other factors. Human experiences vary, and people who experience more harmful events throughout their lives may come to store self-related representations that are more negative. Schemata can be modified and when this happens, learning occurs. When people are not able to assimilate new information because it does not match their schemata, they modify their schemata in order to accommodate the new information.

We know from the literature on depressive realism that people who experience symptoms of mild depression, specifically low mood, tend to adopt more accurate beliefs about themselves (see Bortolotti and Antrobus, 2015, for a recent review). These judgements may include judgements about their own performance, estimation of future self-related events, or memories of the type of feedback they received. Whilst at least in some circumstances people with low mood perceive and assess themselves more accurately than people with no depressive symptoms, people affected by severe forms of depression form negatively biased beliefs about themselves and their circumstances. The more severe depressive symptoms do they have, the more negative self-related beliefs they adopt. Indeed, people who experience major depressive disorder often have critically low self-esteem, perceive themselves as incapable and helpless, are filled with guilt and regrets, and believe that they deserve punishment (Goodwin and Jamison, 1990). They become their most severe critics and their harshest enemies; their thoughts become ruminative and centre on self-condemnation.

## A hypothesis about the development of depressive delusions

Cognitive models share the premise that maladaptive thinking and negative assessments of the self contribute to the development of depression. Aaron Beck’s original theory (Beck, 1967; Beck *et al*., 1979) served as the catalyst for an explosion of research on the so-called ‘cognitive vulnerability to depression’ (Ingram, 1984; Teasdale and Barnard, 1993; Teasdale, 1997; Ingram *et al*., 1998; Hankin and Abramson, 2001; Abramson *et al*., 2002; Beevers, 2005). Some of the most recent cognitive models of depression have involved a refinement and further articulation of the basic model, which has it that maladaptive cognition contributes to the onset of depression in the context of stressful life circumstances (Dozois and Beck, 2008). For our purpose, which is to understand the nature of depressive delusions, it is useful to briefly revisit this model.

Beck (1967) argues that there are three main levels of thinking involved in the onset, maintenance, and aggravation of depression: *depressive self-schemata*, maladaptive beliefs, and negative automatic thoughts. Schemata are central to Beck’s cognitive model. Although they have been defined in a variety of ways, most definitions incorporate the idea that they consist of both structural properties and propositional elements (Ingram *et al*., 1998; Dozois and Beck, 2008). As an organized structure, a schema is often adaptive insofar as it facilitates the speed with which people process information. However, well-organized internal representations are sometimes associated with a cost: the information may be selectively attended to, encoded, and retrieved in a manner that is coloured by one’s internal representation. For instance, a schema of the typical Italian person (emotional, pasta-eating, coffee-drinking, etc.) may give rise to stereotypical, and ultimately incorrect, beliefs about how a group of persons is likely to behave (Linville, 1982).

In the case of depression, the efficiency associated with the use of a schema involves a bias toward attending to, encoding, and retrieving schema-consistent (that is, negative) information about the self, at the expense of positive or neutral information. Previous experience and knowledge structures influence the processing of new information, and self-schemata “are considered dysfunctional in that they embody a constellation of dysfunctional attitudes that lead to negative perspectives about oneself, the world, and the future” (Scher *et al*., 2005, p. 489).

According to Beck, depressive self-schemata develop during early childhood but remain hidden until activated later in life by adverse circumstances (see Beck *et al*., 1979; James *et al*., 2007; Young *et al*., 2003). Leslie who is vulnerable to depression has core beliefs about being fundamentally inept and unlovable. She does not succumb to depression, however, as long as her core beliefs are not *activated*. Once her negative self-schema is triggered by an experience of rejection, Leslie becomes vulnerable to information processing biases (see Scher *et al*., 2005) and experiences negative thoughts that focus on themes of loss, failure, worthlessness, defectiveness, incompetence, and inadequacy (Beck *et al*., 1985; Beck *et al*., 1979).

When depressive self-schemata are activated by negative life experiences, cognitive errors, and negative automatic thoughts follow (Dozois and Beck, 2008). Automatic thoughts refer to the stream of cognitions that arise by association in people’s day-to-day lives and are not accompanied by any direct consideration or volition. Automatic thoughts are more superficial and proximal to the given stimulus than are thoughts at higher levels of cognition, and are functionally related to people’s deeper beliefs. They are the cognitive by-products of activated schemata. Different aspects of people’s core belief system are activated by external environmental cues, or as reactions to internal states and emotions (Dozois and Beck, 2008). These thoughts usually take the form of a negative view of the self, the world, or the future, what Beck *et al*. (1979) called the *cognitive triad*.

To sum up, cognitive models of depression rely on the activation of negative self–schemata triggered by adverse life circumstances. In our example, Leslie adopts increasingly negative beliefs about herself that contribute to her experiencing depressive symptoms. Compatibly with Beck’s cognitive model of depression, one can argue that in depressive illness the process of accommodating new information about oneself in one’s self-schemata is disrupted, and works selectively. People suffering from depression are not capable of successful accommodation, that is, they cannot modify their negative representations of themselves to match new, contradicting information. Because the new information cannot be assimilated in its original form, people distort its content in order to match their self-schemata, and then they assimilate the distorted content. In this way, they compensate for the missing part of the adaptation process, and avoid dissonance.

The empirical literature provides strong evidence in support of this hypothesis. For example, it has been shown that people with depression verify their self-representations by seeking negative – rather than positive – appraisals (Swann *et al*., 1992a). Similarly, people suffering from dysphoria or depression prefer unfavourable evaluations and relationship partners who offer such evaluations (Swann *et al*., 1992b). Although people with depression desire praise, they also seek confirmation for their own negative self-related judgments. Their desire to obtain confirmation for their own negative self-related judgements overrides the desire for praise (Swann, 1990), and their efforts to obtain confirmation intensify when others’ positive appraisals present a challenge to their self-views (Ingram, 2009). Using Festinger’s cognitive dissonance framework, we can say that, as positive appraisals contradict the person’s negative self-schema, they become a source of cognitive dissonance and anxiety. Because of that, they are rejected.

The process goes as follows:

(i) New information becomes available that does not match the existing self-schema.
(ii) The accommodation process is disrupted. Therefore the modification of the schema in order to match the new information is not possible.
(iii) The new information gets distorted in order to be assimilated in the existing negative schema.
(iv) Extremely negative self-related beliefs are adopted.

Here is an example. John sees himself as a bad father. He works long hours and travels on business most weekends. He feels permanently guilty for not giving his children the time they deserve. He brings his children little souvenirs from his trips. The children are always excitedly waiting for his return home and seem to be very grateful for the gifts. However, seeing their joy and love does not change John’s view of himself as a bad father. He believes that the children smile because they were told by their mother to show gratitude. ‘They hate me and one day they will be tired of pretending’ – he thinks.

In the example, John has a negative schema of himself – let us call this ‘a bad father’ self-representation. There is nothing peculiar about it – people have negative representations of themselves that apply to various aspects of their lives. When confronted with contradicting evidence, i.e. the joy and gratitude of his children, John is unable to assimilate the fact that he makes his children happy, as it does not match his ‘bad father’ schema. Unlike many other people, he is also unable to accommodate this new information by modifying his representation of himself. In order to successfully complete the process of adaptation and to maintain coherence, John distorts the new information in order to preserve his negative self-schema and he interprets his children’s joy as an act of pretence.

The process of what we may call *selective* or *distorted* assimilation is repeated every time contradicting data become available. The self-schema expands, enriched with new matching content. The more of the self-related negative content is assimilated into a schema, the more difficult it becomes to modify the schema. The so-called ‘depressive vulnerability’ described by Beck can be seen as a large number of related negative self-schemata. Some people hold negative self-representations that apply to many areas of their lives but do not develop clinical symptoms: this is due to their ability to modify self-representations in order to assimilate new, positive information about themselves. They gradually come to perceive themselves in a better light.

However, accommodation does not work for everybody, and depression emerges as a consequence. Positive information becomes distorted in order to be assimilated in negative self-representations, and thus existing self-schemata expand, enriched with apparently matching content. Negative self-related information is quickly assimilated, because it matches the existing schemata that become more and more articulated. The expansion of negative self-schemata is reflected in the judgments that people with depression express about themselves: ‘I am worthless,’ ‘I have done nothing good in my life,’ ‘I deserve to be punished,’ etc.

Why do people with depression distort new positive information to match their negative self-representations? Living in an almost perpetual mental state of cognitive dissonance resulting from the inability to carry out a successful adaptation would cause overwhelming stress and anxiety. Judgements such as ‘I am worthless’ keep the anxiety caused by cognitive dissonance at bay, but may be another source of anxiety due to their content which emphasises loss, deprivation, self-deprecation, and hopelessness (Beck, 1967; Beck *et al*., 1979; Dozois and Beck, 2008). A person who experiences severe depression resolves cognitive dissonance but only temporarily: John’s negative self-appraisal of himself as a bad father, and his sense of guilt, lead him to expect lack of affection and detachment from his children, which he believes he deserves. However, as his ‘bad father’ beliefs do not necessarily represent reality accurately, the reaction he expects is not observed, and the coherence of his self-representation is under threat again.

## Psychological and epistemic benefits of depressive delusions

According to the ECS model, preserving consistency is a priority for human cognition (Festinger, 1957, 1962; Brehm and Cohen, 1962; Abelson, 1968; Elliot and Devine, 1994; Goetzmann and Peles, 1997). People have a general tendency to restore coherence between their views, and between their views and the views of others, when this is compromised (Heider, 1946). If a person finds herself in a state of extreme cognitive dissonance, she needs to validate her prior beliefs and offset the cognitive dissonance emerging from those beliefs and newly acquired information. Mood-congruent delusions in depression play this key role. In particular, they may offer validation for one’s own intensively experienced guilt, shame, hopelessness, and dismay.

The claim that one is being spied upon in one’s own home – for example –matches the belief that one is not trust-worthy and should be monitored at all times. Undoubtedly such delusions come with psychological costs. The delusional content is weaved with unpleasant, sometimes terrifying events, such as being watched, followed, or threatened. But, by reducing dissonance, delusions also offer psychological relief from dissonance–related anxiety. The delusion–induced anxiety that replaces the dissonance-related anxiety has the advantage of reinstating consistency: the negative beliefs about the self are confirmed.

Is the hypothesis that depressive delusions validate and confirm prior beliefs about the self compatible with the prediction-error theory of delusion formation (Fletcher and Frith, 2009; Corlett *et al*., 2007; Griffiths *et al*., 2014) which has already been applied to delusions in schizophrenia? According to the model proposed by Philip Corlett and colleagues for schizophrenic delusions, delusions are formed in response to aberrant prediction-error signals, those signals that indicate a mismatch between expectation and actual experience (Miyazono *et al*., 2014). A prediction error happens when new incoming information does not match the person’s existing representations (schemata) and, therefore, cannot be successfully integrated in the person’s model of the world (assimilated). It indicates that the internal model of the world from which the prediction is derived is incorrect and needs to be updated. By updating the model in such a way as to minimize prediction errors, the person gains a better understanding of the world. In this framework, prediction-error signals play a fundamental role in learning.

In a recent reconstruction of the stages of delusion formation in schizophrenia (Mishara and Corlett, 2009), three stages are identified:

(i) *Anxious expectation*, when the agent bombarded with prediction-error signals is constantly expecting something important to happen and the processes underlying automated and habitual learning are disrupted.
(ii) *Revelation*, when the delusion is formed putting an end to overwhelming anxiety and to the sense of unpredictability. The events previously experienced as inexplicable and distressing no longer require attention and the processes underlying automated and habitual learning resume their normal function. The person’s model of the world has been updated to include the delusion.
(iii) *Reinforcement*, when the delusion is stamped into the agent’s memory and reinforced every time the previoulsy inexplicable events are experienced.

Prediction-error theorists argue that abnormal prediction-error signaling contributes to the formation of delusions (Miyazono *et al*., 2014). In particular, they hypothesize that in people with delusions prediction-error signals are excessive and they are produced when there is no real mismatch between expectation and experience. The excessive prediction-error signals falsely indicate that the person’s internal model of the world needs to be updated even though, as a matter of fact, it does not have to be.

In the case of depressive delusions, the process differs as people err on the side of *conservatism* as opposed to *revisionism*. Instead of changing their representation of the world to match the unusual experience, validating new experience at the expense of previously acquired beliefs, people with depressive delusions preserve their representation of themselves despite the new information that conflicts with it, validating the existing self-schemata at the expense of the new information that is reinterpreted and distorted to fit the schema. When this happens, in the language of the ECS model, an adaptation process has to occur for the system to regain cognitive balance. If the person is unable to update her internal model on the basis of the incoming data, she will reinterpret and distort the content of the data in order to match the schema. The delusions that emerge are mood-congruent and bringing validation to the original, negatively biased, self-representation.

In the prediction-error model described by Corlett and colleagues, the adoption of delusions in schizophrenia has some positive contribution to make and delusions should not be seen exclusively as a deficit. In particular, the claim is that, in the context of the disruption of the learning process caused by excessive prediction-error signals, delusions “permit continued engagement with an overwhelming world, and ongoing function in the face of paralyzing difficulty” (Fineberg and Corlett, 2016, p. 73). The claim makes reference to the notion of *epistemic innocence* that has been applied to motivated delusions and elaborate and systematised delusions in schizophrenia (Bortolotti, 2015, 2016). Can this notion be applied also to depressive delusions?

Epistemically innocent cognitions are not necessarily free from epistemic faults, but they do have significant epistemic benefits that would be unattainable otherwise (Bortolotti, 2015, p. 495). Our suggestion is that, if the adoption of a delusional hypothesis helps avoid bad epistemic consequences and adopting another (non-delusional) hypothesis would not have the same benefit, then the adoption of the delusional hypothesis is an acceptable response to what we can view as an emergency situation.

Here are the two conditions for the epistemic innocence of delusions:

1. *Epistemic benefit*: The adoption of the delusional hypothesis confers a significant epistemic benefit to a given agent at a given time.
2. *No alternatives*: Alternative hypotheses that would confer the same benefit are not available to that agent at that time.

Some qualifications are in order. First, what counts as an epistemic benefit may vary. One might say that adopting a delusion is epistemically beneficial if it contributes to the acquisition or retention of true beliefs, if it promotes the agent’s intellectual virtues, or if it is something an agent should be praised and not blamed for. One’s commitments in epistemology will affect the way in which the epistemic benefits are identified and described. In the case of depressive delusions, the main benefit seems to be the preservation of a coherent self-representation.

Second, different notions and degrees of unavailability can explain the failure to adopt a less epistemically costly hypothesis. This spectrum of possibilities reflects the nature of the limitations that the agent experiences in the relevant context, ranging from standard reasoning limitations affecting all human agents to deficits of perception, inference, or memory that may apply in clinical settings. For instance, with respect to delusions in schizophrenia, one may suggest that in the person who adopts a delusional hypothesis there is an impairment in the capacity to evaluate hypotheses on the basis of their plausibility before adopting them as beliefs (this is an idea developed in the context of the two-factor theory of delusion formation, as in Davies *et al*., 2001). Alternatively, one may suggest that the person is so overwhelmed by the delusional hypothesis that she does not even consider alternative interpretations of her unusual experience (as in Freeman *et al*., 2004). In the case of depressive delusions, the unavailability of alternative hypothesis is driven by the process of negatively-biased learning we described earlier: people have long ignored positive information about themselves or re-interpreted it with a negative spin, and this has not been integrated in their self-schemata.

Third, epistemic innocence applies to the *adoption*, not the prolonged *maintenance*, of delusional beliefs. The benefit consists in avoiding a problem that presents itself when the unusual experience or the new evidence is not yet made sense of, and can generate anxiety, stress, tension. Delusions appear as a response to a critically high point (a ‘tipping point’) of cognitive imbalance (or, quoting Jaspers, a ‘limit situation’), and their function is to reinstate the balance that has been lost. From an epistemic standpoint, by reducing dissonance and preventing the disintegration of the self, depressive delusions help restore the person’s narrative identity. With reference to our previous example, John can preserve a concept of himself as a bad father, despite the reassurances of his wife and the gratitude expressed by his children, by distorting evidence that he is a good, highly appreciated, father. For the person who seeks consistency between her self-related beliefs and feelings and newly acquired information, the depressive delusion offers a solution. But the benefit is temporary. When the delusion becomes entrenched, as we showed both in the case of schizophrenic and depressive delusions, it becomes a new source of stress, anxiety, and tension.

To sum up, the most important function of depressive delusions seems to be that they restore the equilibrium between the cognitive processes responsible for adaptation. Depressive delusions contribute to the agent’s narrative identity by validating prior self-related beliefs and feelings that happen to be negatively biased. They can be considered as epistemically innocent, which means that they deliver significant epistemic benefits which could not be achieved otherwise.

## Conclusions

In this paper we asked what depressive delusions are and whether they have the potential for psychological and epistemic benefits. We argued that depressive delusions emerge as a result of the attempt to eliminate the inconsistency between self-schemata formed via a biased process of learning, and new conflicting information. Their function is to restore the equilibrium of the cognitive processes of adaptation, assimilation, and accommodation. New information is distorted in order to be assimilated and integrated in previously acquired self-schemata, which consist of negatively biased self-related beliefs and feelings. Due to their reducing dissonance and providing the basis for a unified narrative self, depressive delusions have the potential to deliver both psychological and epistemic benefits by relieving dissonance-induced anxiety and preserving a coherent self-concept. But in the long run, their distressing content causes serious psychological harm, and the mounting evidence against the self-schemata they are designed to preserve is destined to compromise the person’s delicate cognitive balance.

In this paper we have not offered an exhaustive account of delusions in depression, but highlighted the need for more research into this fascinating and under-studied phenomenon. Our preliminary consideration of the literature suggests that the experience of depressive delusions is on a spectrum with everyday experiences of attempting to maintain a previously acquired belief by reinterpreting new evidence that conflicts with it, and thus makes the cognitive mechanisms responsible for depressive delusions continuous with those underlying non-pathological beliefs. A better understanding of depressive delusions can offer some insights into the challenges faced by a person with a major depressive episode accompanied by psychotic symptoms, and pave the way for interventions that respect the complexity of the delusional experience, both its highly distressing long-term effects and its temporary adaptive features.
